# Intravenous Immunoglobulin (IVIG) for Patients with Severe Neurotoxicity Associated with Chimeric Antigen Receptor T-Cell (CAR-T) Therapy

**DOI:** 10.3390/ijms26083904

**Published:** 2025-04-21

**Authors:** Sepideh Mokhtari, Justin M. Asquith, Syeda Saba Kareem, Christina A. Bachmeier, Yolanda Pina, Rawan G. Faramand, Youngchul Kim, Edwin N. Peguero, Solmaz Sahebjam, Mohammad H. Jaffer, David P. Iacono, Michael D. Jain, Michael A. Vogelbaum, Marco L. Davila, Peter A. Forsyth, Frederick L. Locke, Aleksandr Lazaryan

**Affiliations:** 1 Department of Neuro-Oncology, Moffitt Cancer Center, Tampa, FL 33612, USAedwin.peguero@moffitt.org (E.N.P.); peter.forsyth@moffitt.org (P.A.F.); 2 Department of Medical Oncology, Aspirus Cancer Care, Wausau, WI 54401, USA; 3 Department of Bone Marrow Transplant and Cellular Immunotherapy, Moffitt Cancer Center, Tampa, FL 33612, USA; 4 Department of Biostatistics, Moffitt Cancer Center, Tampa, FL 33612, USA; 5 Department of Medical Oncology, John Hopkins Hospital, Washington, DC 20016, USA; 6 Department of Medicine, Roswell Park Comprehensive Cancer Center, Buffalo, NY 14203, USA

**Keywords:** CAR-T, neurotoxicity, IVIG, CRES, axicabtagene ciloleucel, ICANS, CAR-T-related encephalopathy syndrome

## Abstract

Severe immune effector cell-associated neurotoxicity syndrome (ICANS) occurs in about 30% of all patients with large B-cell lymphoma (LBCL) who are treated with axicabtagene ciloleucel (axi-cel). There are currently limited treatment strategies other than the standard corticosteroids, and it is essential to find additional therapies to manage severe ICANS. We conducted a retrospective study of neurologic outcomes among patients who received axi-cel for LBCL from May 2015 to February 2019. We identified patients who developed severe ICANS and were treated with glucocorticoids followed by intravenous immunoglobulin (IVIG) (n  =  9) or glucocorticoids alone (n  =  10). There was no statistically significant difference in the time to resolution (TTR) of severe ICANS between groups; however, patients in the IVIG had more severe grades of ICANS with a lower performance status at baseline. The cumulative steroid days were 11.2 in the IVIG arm and 13.5 in the glucocorticoids-only arm. The use of IVIG for severe ICANS after axi-cel therapy was tolerable and safe and is generally recommended in the CAR-T setting in patients with hypogammaglobinemia. The use of IVIG as a potential therapeutic agent for severe ICANS can be further explored in future prospective studies.

## 1. Introduction

For patients with refractory LBCL, adoptive therapy with T-cells genetically engineered to express a CD19 chimeric antigen receptor (CAR) [1] leads to objective response rates ranging from 64% to 83% and complete response rates ranging from 43% to 58% [2,3]. To treat relapsed/refractory LBCL after two prior lines of therapy, the US Federal Drug Administration (FDA) has approved several CAR-T-cell therapies, including axi-cel tisagenlecleucel and lisocabtagene maraleucel.

However, CAR-T-cell therapy can cause cytokine release syndrome (CRS) among 37−93% of patients [4] and ICANS [5] among 23–67% of patients [4,6]; severe ICANS (grade ≥ 3) occurs among approximately 30% of patients [2]. The median time to onset of ICANS is usually 5 to 7 days after axicabtagene ciloleucel anti-CD19 CAR-T-cell (axi-cel) infusion, as noted in the ZUMA trials [7]. Current guidelines recommend using tocilizumab, an anti-interleukin- (IL-) 6 receptor therapy, to treat refractory CRS or concurrent CRS with ICANS; however, it is not used to treat severe ICANS without CRS symptoms. Glucocorticoids are recommended to treat grade ≥ 2 ICANS [7,8]. There are limited options to manage steroid-refractory ICANS, and other therapies that are being studied include anakinra, an IL-1 antagonist [8].

The pathophysiology of ICANS is not completely understood, but emerging evidence suggests that CAR-T-induced inflammation triggers endothelial cell activation, resulting in a breakdown of the blood–brain barrier (BBB) [9], elevated cerebrospinal fluid (CSF) cytokine levels, and T-cell accumulation in the CSF/brain parenchyma, all of which are consistent with non-infectious encephalitis [10]. One case series of 20 patients with ICANS reported an elevation of CSF protein levels, white blood cell (WBC) count, cytokines, and excitatory neurotransmitters [11]. These findings suggest that a surge of cytokines and inflammatory cells in the CSF accelerates central nervous system (CNS) inflammation, thereby triggering ICANS.

IVIG effectively treats inflammatory neurologic disorders, including limbic encephalitis [12]. IVIG works by suppressing macrophage-mediated demyelination [13], increasing the ratio of FcγRII/FcγRIII receptors on monocytes [14], suppressing myeloid cells [15], increasing CD4 T-cell infiltration and activation in the CNS [16], and reducing circulating levels of IL-6, IL-8, interferon- (IFN-) γ, and granulocyte-macrophage colony-stimulating factor (GM-CSF) [17,18]; IVIG has also been known to cross the BBB [19]. In the setting of CAR T-cell therapy, IVIG is used in patients with hypogammaglobulinemia and with recurrent infections [19]. Given CAR T-cell therapy’s proposed immune-mediated mechanism of neurotoxicity, we hypothesize that IVIG may be a beneficial and safe treatment option for patients experiencing severe ICANS following unsuccessful use of glucocorticoids.

## 2. Results

A total of 19 (26%) adult patients out of 72 recipients of axi-cel therapy with LBCL developed severe ICANS within 30 days of axi-cel infusion. A total of 9 of 19 patients received glucocorticoids + IVIG and 10 received glucocorticoids only. Table 1 summarizes the clinical features of patients in each group. The median age of the entire cohort was 63 years (range, 47–75), and 68% of patients were male. The glucocorticoids + IVIG and glucocorticoids-only groups had similar International Prognostic Index (IPI) scores (*p* = 0.63), LBCL stages (*p* = 0.3), rates of bulky disease >10 cm (*p* = 0.14), and time to onset of severe ICANS (median, 6 days in both groups; *p* = 0.43). However, pre-CAR-T therapy Eastern Cooperative Oncology Group (ECOG) performance status was on average significantly worse among patients who subsequently required glucocorticoids + IVIG (*p* = 0.03).

**Figure 1:**
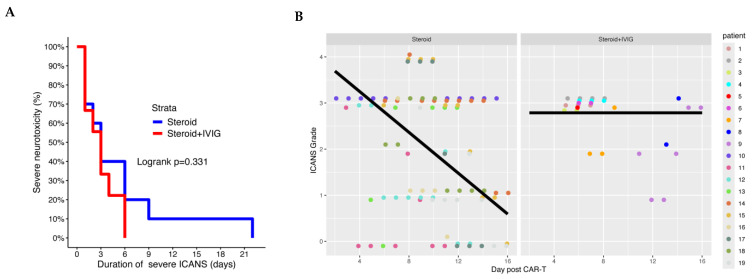
(**A**): Kaplan–Meier curve of time to resolution of severe ICANS. Abbreviations: ICANS, immune effector cell-associated neurotoxicity syndrome; IVIG, intravenous immunoglobulin. (**B**): Change in neurotoxicity grade over time. Glucocorticoids-alone group: change in ICANS grade for patients after receiving glucocorticoids alone. Steroid + IVIG: change in ICANS grade for patients who received IVIG + glucocorticoids after the initiation of glucocorticoids and before the administration of IVIG. Abbreviations: CAR-T, chimeric antigen receptor T-cell; CI, confidence interval; ICANS, immune effector cell-associated neurotoxicity syndrome; IVIG, intravenous immunoglobulin.

The values for cumulative steroid days were 11.2 (range, 4–26) in the glucocorticoids + IVIG arm and 13.5 (range, 6–33) in the glucocorticoids-only arm. The median cumulative steroid days after initiation of IVIG was 7.77 days (range, 2–21). The median TTR of severe ICANS down to grade ≤ 2 while receiving glucocorticoids was 3 days for both groups (log-rank *p*  =  0.33; Figure 1A).

**Figure 2:**
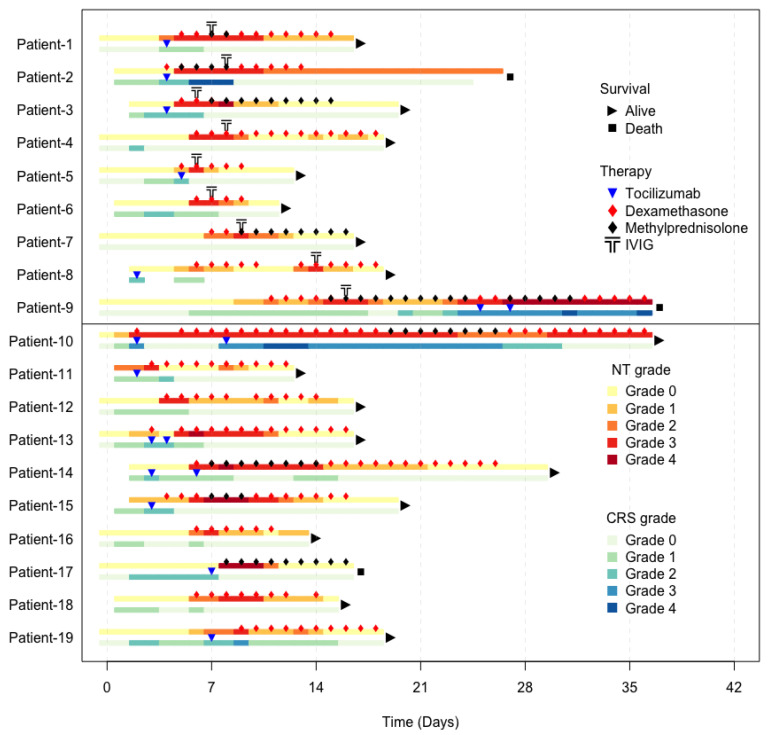
Clinical outcomes and medical interventions after CAR-T therapy in patients over time. Patients 1 through 9 received IVIG + glucocorticoids and patients 10 through 19 received glucocorticoids alone. The top bar represents the ICANS profile, and the lower bar represents the CRS profile. The grading of each type of toxicity is labeled with a different color, as demonstrated in the legend. Each intervention, such as tocilizumab, dexamethasone, methylprednisolone, or IVIG, is labeled with a different symbol, as demonstrated in the legend. Abbreviations: CAR-T, chimeric antigen receptor T-cell; CRS, cytokine release syndrome; ICANS, immune effector cell-associated neurotoxicity syndrome; IVIG, intravenous immunoglobulin; NT, neurotoxicity.

Upon the administration of the first dose of IVIG, the median TTR of severe ICANS was 1 day (range, 1–4 days). The median time to administration of IVIG after CAR-T infusion was 8 days (range 6–15). The median time to administration of IVIG after severe neurotoxicity and initiation of glucocorticoids was 1 day (range, 0–3 days). The objective response rate at 30 days was 78% among patients who received glucocorticoids + IVIG vs. 80% among patients who received glucocorticoids alone (*p* = 0.91). Three patients (two from the glucocorticoid + IVIG group and one from the glucocorticoid group alone) died within 40 days of axi-cel therapy. Causes of death included lymphoma progression (n  =  1), hemophagocytic lymphohistiocytosis (n  =  1), and hemorrhagic stroke (n  =  1). No patients who received glucocorticoids + IVIG developed thromboembolism, renal failure, or autoimmune hemolytic anemia. Patient-level data on serial changes in ICANS/CRS grades, clinical outcomes, and medical interventions are summarized in Figure 2.

Most patients who received glucocorticoids + IVIG had persistent or worsening ICANS grades at least 48 h after the initial administration of glucocorticoids, and these patients were subsequently treated with IVIG. The change in ICANS grade over time after the administration of axi-cel therapy, before the initiation of IVIG in the glucocorticoids + IVIG group and the first 2 weeks after administration of axi-cel therapy in the glucocorticoids-alone group, was assessed (Figure 1B). The linear regression slope was −0.018 (95% CI, −0.199–0.163) for patients who received glucocorticoids + IVIG and −0.221 (95% CI, −0.312–−0.131) for patients who received glucocorticoids alone (*p* = 0.049). This demonstrates that patients in the glucocorticoids + IVIG group were sicker before the initiation of IVIG compared to patients who received glucocorticoids alone.

**Figure 3:**
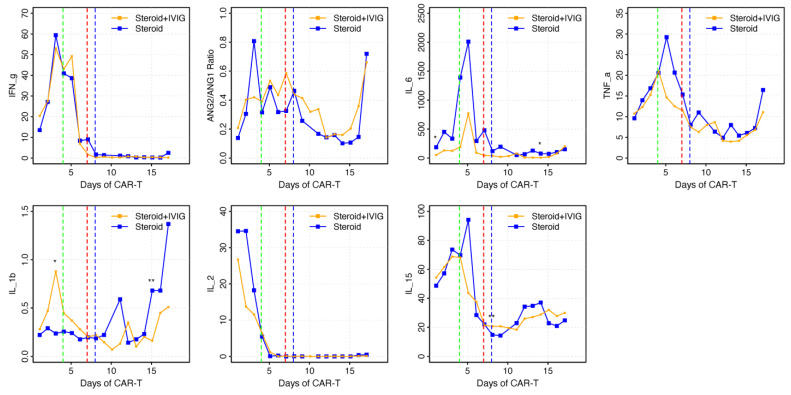
Comparison of median cytokine levels in each group. Serum cytokine data were available for 7 patients who received IVIG + glucocorticoids and 4 patients who received glucocorticoids alone. Dashed lines represent the median day each intervention was given after CAR-T therapy. Green dashed line: tocilizumab (day 4). Red dashed line: glucocorticoids (day 7). Blue dashed line: IVIG (day 8). * trend, but not significantly different (*p* < 0.1); ** significant difference (*p* < 0.05). Abbreviations: CAR-T, chimeric antigen receptor T-cell; IVIG, intravenous immunoglobulin.

Serum cytokine data were only available for seven patients (Figure 3) who received glucocorticoids + IVIG and for four patients who received glucocorticoids alone. Overall, both groups had similar elevations of IL-2, IL-6, IL-15, IFN-γ, tumor necrosis factor- (TNF-) α, and angiopoietin (Ang-2/Ang-1) ratio following axi-cel therapy and preceding any therapeutic interventions for ICANS or CRS. Baseline immunoglobulin G (IgG) levels < 400 mg/dL were documented among 25% of patients in the glucocorticoids + IVIG group and 33% among the glucocorticoids-only group.

## 3. Discussion

According to many guidelines, IVIG can be considered for patients with hypogammaglobulinemia with IgG < 400 mg/dL and a high risk of infections after CAR T-cell therapy [5,8]. Our study is the first, to the best of our knowledge, to study the use of IVIG in the management of severe ICANS concurrently with glucocorticoids in comparison with outcomes following the use of glucocorticoids alone. The use of IVIG to treat severe ICANS after axi-cel therapy appeared to be safe and tolerable in patients with LBCL. Although our analysis demonstrated no significant difference in TTR for severe ICANS among patients who received glucocorticoids + IVIG vs. glucocorticoids alone, it demonstrated that the patients who received glucocorticoids + IVIG had significantly worse performance status as assessed by the ECOG scores before CAR-T therapy and also persistent or worsening ICANS. Both of these observations could have contributed to a longer duration for the resolution of severe ICANS in the glucocorticoids + IVIG group.

Prospective controlled studies with larger sample sizes are needed to further clarify the potential efficacy of IVIG for treating severe ICANS after CAR-T therapy refractory to standard-of-care steroids for the first 48 h following administration. It remains unclear if any immune-modulating interventions used to treat CAR-T toxicity interfere with the efficacy of CAR-T therapy. We found no clear evidence that any toxicity management strategies, such as steroids, tocilizumab, or IVIG, affected CAR-T’s efficacy against LBCL. The objective response rates for both groups appear to be within range of those reported in the pivotal trials for CAR-T for LBCL [2,20], especially for patients with severe ICANS who do not experience timely responses and continue to deteriorate, resulting in prolonged hospitalization, excessive morbidity, and occasional mortality. Prospective clinical trials are warranted to definitively determine whether the therapeutic efficacy of CAR-T can be maintained with the use of IVIG, glucocorticoids, or both.

Though our data provided valuable insights into responses to IVIG among patients with severe ICANS, the precise mechanism of action for IVIG to treat ICANS remains unknown. A non-human primate model of ICANS demonstrated an elevation of CSF cytokines, such as IL-6, IL-2, GM-CSF, and vascular endothelial growth factor (VEGF), together with CAR-T and non-CAR-T-cell accumulation in the CSF and brain parenchyma, similar to that seen in non-infectious encephalitis [10]. In our small cohort, both groups had overall similar cytokine level patterns associated with elevations of IL-2, IL-6, IL-15, IFN-ϒ, TNF-α, and Ang-2/Ang-1 ratio before any therapeutic interventions for ICANS or CRS. However, the cytokine levels were most likely influenced by the administration of steroids, tocilizumab, and IVIG.

We did not find a clear benefit from the reduction in serum cytokines that are known to be associated with ICANS after the administration of IVIG. However, we did not have sufficient data to compare CSF cytokine characteristics between the two subgroups. IL-1β and TNF-α have been shown to induce ICANS through glutamate production by inducing neuronal glutaminase [21], which is the predominant glutamine-utilizing and glutamate-producing enzyme in neurons. This enzyme has the potential to elevate glutamate to an excessive level and cause neurotoxicity. There is some evidence that IVIG could selectively inhibit IL-1β- and TNF-α-dependent neutrophil transepithelial migration across epithelial cells [22]. Santomasso et al. also identified elevated glutamine agonist levels in CSF during neurotoxicity after CAR-T therapy among patients with acute lymphoblastic leukemia [11].

We also found an increased median ratio of Ang-2 to Ang-1 in the first 7 days following CAR-T therapy. A higher Ang-2/Ang-1 ratio has been shown to indicate a BBB breakdown and increased vascular damage [9]. Many studies have found that an increased Ang-2/Ang-1 ratio is associated with an increased risk of developing grade 4 ICANS [23]. Higher GM-CSF levels have also been linked with an increased ICANS grade, and therefore, GM-CSF suppression by IVIG, which contains natural anti-GM-CSF antibodies, can serve as an additional mechanism of action for IVIG to treat severe ICANS [18]. IVIG-mediated inhibition of critical CNS cytokines or neurotransmitters can also contribute to its potential efficacy.

The safety profile of patients in the glucocorticoids + IVIG group is worth noting, as this was a well-tolerated intervention. No patients developed any IVIG-induced complications, such as renal failure, thromboembolism, or hemolytic anemia. This safety profile should be carefully considered within the larger context of determining the best ICANS management practices, as consensus guidelines are rapidly evolving. These guidelines need to be refined, as CAR-T therapy has been FDA-approved and toxicity recommendations may be widely adapted for many tumor types and specific immune effector therapies. Current consensus guidelines recommend glucocorticoids as an initial treatment for ICANS. Other treatment alternatives that are still being studied for this population include siltuximab, an anti–IL-6 antibody, and anakinra, a recombinant humanized IL-1 receptor antagonist. A retrospective study by Werli et al. on 14 patients with steroid-refractory ICANS showed statistically significant and rapid reductions in fever, inflammatory cytokines, and biomarkers associated with ICANS/CRS after anakinra treatment [24]. However, anakinra did not have a significant effect on ICANS or the rapid tapering of glucocorticoids. The cumulative steroid days seen in that study was 12.5 days; however, in our study, 11.2 days was seen in the IVIG group versus 13.5 days in the steroid-only group.

Our pilot study was limited to a small sample size of 19 patients who developed severe ICANS and received different interventions for severe ICANS management. Therefore, the study did not have enough power to detect significant differences in TTR between the two groups. We need larger cohorts in future studies to assess the statistically significant differences between the two groups. Another limitation of the study is its selection bias, which is inherent in this type of retrospective analysis. Patients in the glucocorticoids + IVIG group, compared to the average LBCL patient population, tended to have more severe disease, had higher ECOG scores, more frequently had worsening or persistent ICANS despite initial glucocorticoid treatment, and required an administration of IVIG. We, therefore, were somewhat limited in our ability to fully differentiate the efficacy of IVIG intervention from a delayed effect of steroids. We need larger cohort studies comparing patients with similar characteristics and only exposure to glucocorticoids or IVIG to better assess the efficacy of IVIG. Furthermore, treatment decisions regarding IVIG administration can be subjective; however, only two neurologists were involved in the management of severe ICANS for all 19 patients reported here, thereby limiting any substantial decision-making heterogeneity.

In summary, severe ICANS associated with axi-cel therapy may lead to life-threatening complications. Despite more rapidly rising ICANS grades among patients who received IVIG, the TTR of severe ICANS was similar among recipients of glucocorticoids + IVIG vs. recipients of glucocorticoids alone. The cumulative steroid days were shorter in the glucocorticoids + IVIG group; however, larger prospective, randomized, controlled clinical trials are needed to further explore the efficacy of IVIG in treating severe ICANS. Patients with hypogammaglobinemia could additionally benefit from this management strategy, as administration of IVIG for this population was feasible, and based on our study, it is a safe and tolerable treatment option without any severe side effects.

## 4. Materials and Methods

We conducted a single-center retrospective cohort study of neurologic and oncologic outcomes among two groups of patients who received axi-cel infusion to treat LBCL and subsequently developed grade ≥ 3 ICANS: those who were treated with glucocorticoids followed by IVIG and those who were treated with glucocorticoids only. We obtained Investigational Review Board (IRB) approval for this observational study. The Moffitt Cancer Center Immune Effector Cell database was searched for patients who received axi-cel infusion to treat LBCL or its variants between November 2017 and February 2019. We further identified patients who developed severe ICANS within 30 days of axi-cel therapy. We then studied patients who developed severe ICANS and were treated with glucocorticoids + IVIG (n  =  9) or glucocorticoids alone (n  =  10).

Clinical, laboratory, electrodiagnostic, radiological, pathological, and serum cytokine data were analyzed for each patient. Clinical follow-up was from the time of administration of axi-cel up to 6 months after the administration of axi-cel, and the time to resolution (TTR) of severe ICANS was defined as the time from glucocorticoid initiation until the improvement of ICANS to grade ≤ 2. ICANS was graded using the CAR T-cell-related encephalopathy syndrome (CRES)/CAR T-cell-therapy-associated toxicity (CARTOX) grading scale, and CRS was graded per the modified Lee criteria. Severe CRS or ICANS was defined as grade ≥ 3 toxicity by their respective scales [5].

Per institutional guidelines, all patients received prophylactic levetiracetam (750 mg orally twice a day), starting one day before axi-cel infusion; antiepileptic drug regimens were adjusted based on electroencephalogram (EEG) changes and clinical status. The initial management of ICANS at our institution includes 10 mg of intravenous dexamethasone every 6 h; in the case of concurrent CRS, patients are also given 8 mg/kg of tocilizumab. Per institutional guidelines, high-dose methylprednisolone (1000 mg/day) could also be used for grade 4 ICANS.

Demographic and clinical variables were descriptively summarized using continuous and categorical data analyses. Wilcoxon rank-sum and Fisher’s exact tests were used to assess differences in continuous and categorical variables between the patient groups, respectively. The TTR of severe ICANS was analyzed by the Kaplan–Meier method and log-rank test [25]. Serial changes in ICANS/CRS grades after axi-cel therapy were compared between the groups via linear mixed-model analysis. Distributions of total and average daily doses of study therapeutics were depicted using box plots and compared via the Wilcoxon rank-sum test.

## Figures and Tables

**Table 1:** Clinical characteristics of patients.

	All (N = 19)	IVIG (N = 9)	Glucocorticoids (N = 10)	*p* Value
Age: median (range)	63 (47–75)	61 (48–75)	64 (52–74)	0.19
<65 (%)	68	78	60	
≥65 (%)	32	22	40	
Male/female	13/6	6/3	7/3	0.99
Time to severe ICANS ^a^, median days (range)	6 (2–15)	6 (5–15)	6 (2–9)	0.43
ECOG ^b^ performance status				0.03
0–1 (%)	79	55	100	
2–3 (%)	21	45	0	
IPI score				0.63
1–2 (%)	32	78	40	
3–4 (%)	68	22	60	
Stage				0.303
I/II (%)	26	11	40	
III/IV (%)	74	89	60	
% with bulky disease ^c^	11	44	10	0.14
CRS > 1 (%)	68	78	60	0.63
% received Tocilizumab	68	67	70	0.99
IVIG start after CAR-T infusion: median days (range)		8 (6–15)		
Total cumulative steroid dose (mg) ^d^: median (range)		717 (130–2242)	629 (168–1587)	0.37
Cumulative steroid dose after IVIG (mg) ^d^: median (range)		457 (70–1732)		
Total cumulative steroid days: median (range)		11.2 (4–26)	13.5 (6–33)	0.49
Cumulative steroid days after IVIG: median (range)		7.77 (2–21)		

^a^ Onset of severe NT (≥3 grade) before steroid +/− IVIG was implemented; ^b^ pre-CAR-T treatment; ^c^ >10 cm; ^d^ dexamethasone equivalent. Abbreviation: CAR-T, Chimeric Antigen Receptor T-Cell therapy; ECOG, Eastern Cooperative Oncology group; ICANS, immune effector cell-associated neurotoxicity syndrome; IVIG, intravenous immunoglobulin; N, number; IPI, Lymphoma International Prognostic Index.

## Data Availability

Data is contained within the article.
